# Psychosocial Factors and Disease Localization as Independent Predictors of Sexual Dysfunction in Women with Endometriosis

**DOI:** 10.3390/jcm14217788

**Published:** 2025-11-02

**Authors:** Paula Norinho, Rosa Zulmira Vaz de Macedo, Mariana M. Martins, Hélder Ferreira

**Affiliations:** 1 Centre for Psychology, Faculty of Psychology and Education Science, University of Porto, 4200-135 Porto, Portugal; mmartins@fpce.up.pt; 2 Department of Women and Reproductive Medicine, Centro Materno Infantil do Norte Albino Aroso, Centro Hospitalar Universitário de Santo António, 4099-001 Porto, Portugal; rosazulmira@gmail.com (R.Z.V.d.M.); helferreira@hotmail.com (H.F.); 3 Institute of Biomedical Sciences Abel Salazar, University of Porto, 4050-313 Porto, Portugal; 4 Faculty of Psychology and Education Science, University of Porto, 4200-135 Porto, Portugal

**Keywords:** endometriosis, sexual dysfunction, deep infiltrating endometriosis (DIE), Enzian classification, psychosocial factors

## Abstract

**Background:** This study examines the impact of endometriosis on sexual function, focusing on disease localization, pain severity, and psychosocial factors. It integrates the rASRM and Enzian classification systems to explore anatomical contributions to sexual dysfunction. **Methods:** In a cross-sectional study, 102 patients with confirmed endometriosis completed an online evaluation. Of these, 77 had surgical and histological confirmation, and 25 had no prior surgery. Thirty-five participants were using hormonal therapy. Validated instruments assessed sexual function, pain intensity (VAS), and psychosocial variables. Analyses included univariate tests and multivariate logistic regression. **Results:** Deep infiltrating endometriosis involving the sacrouterine ligaments, cardinal ligaments, and pelvic sidewall (Enzian B) was associated with sexual dysfunction, highlighting the anatomical utility of the Enzian system. Surprisingly, those with sexual dysfunction reported lower pain scores (*p* = 0.024 *). In multivariate analysis (R^2^ = 0.281), no individual factor, including Enzian B involvement or EHP scores, remained significant. No associations were found between sexual dysfunction and anxiety, depression, stress, or relationship satisfaction, though pain was inversely correlated with anxiety (*p* = 0.025 *). **Conclusions:** Sexual dysfunction in endometriosis appears multifactorial, not solely driven by lesion burden or pain. A multidisciplinary approach is recommended, addressing anatomical, psychological, and behavioral dimensions.

## 1. Introduction

Endometriosis is a chronic, estrogen-dependent inflammatory condition characterized by the presence of endometrial-like tissue outside the uterus [1,2]. Affecting up to 10% of individuals of reproductive age and up to 50% of those with chronic pelvic pain or infertility, it is one of the most prevalent gynecological disorders [3]. Clinical symptoms include dysmenorrhea, chronic pelvic pain, deep dyspareunia, and infertility [4]. Dyspareunia refers to persistent or recurrent genital pain during sexual intercourse, most commonly during deep penetration. It is important to distinguish dyspareunia from other pain disorders such as vaginismus, which involves involuntary contraction of the pelvic floor muscles that can make penetration difficult or impossible, and vulvodynia, which is defined as chronic vulvar pain without an identifiable cause. These distinctions are essential both for clinical practice and for accurately interpreting research findings on sexual function in individuals with endometriosis [2]. Although surgical and hormonal treatments may alleviate symptoms, particularly dyspareunia [5,6,7], long-term outcomes remain suboptimal [8]. Complex pain mechanisms, including central sensitization, pelvic floor dysfunction, and neurogenic inflammation, can contribute to persistent and hormone-independent pain [9,10].

Accurate classification of endometriosis is critical for guiding treatment. The revised American Society for Reproductive Medicine (rASRM) staging system is widely used but offers limited information on deep infiltrating endometriosis (DIE), which is strongly linked to deep dyspareunia [11,12,13]. In contrast, the Enzian classification provides more detailed anatomical mapping, particularly in surgically relevant regions like the uterosacral ligaments and vagina [14]. Despite its clinical potential, Enzian remains underutilized in sexual function research.

Dyspareunia affects 32–70% of individuals with endometriosis and is influenced by both biological and psychosocial factors [15,16,17]. Anxiety, depression, and maladaptive coping strategies can exacerbate pain perception and impair sexual well-being [18,19,20,21,22,23]. The condition also affects partners, introducing emotional strain and altered relational dynamics [24,25,26,27,28,29,30]. Social pressures, including fear of infertility or partner loss, may lead individuals to downplay symptoms or prioritize relational harmony over personal comfort [28,31].

This study aims to evaluate sexual function in individuals with endometriosis by examining (1) associations with disease localization using rASRM and Enzian classification systems; (2) independent effects of psychosocial factors such as anxiety, depression, and relationship satisfaction; and (3) possible moderating effects of these variables. To our knowledge, this is the first study to integrate both detailed anatomical classifications and psychosocial dimensions in the assessment of sexual function in endometriosis.

## 2. Materials and Methods

This descriptive cross-sectional study was conducted at the tertiary endometriosis center of Centro Hospitalar Universitário de Santo António in Porto, Portugal. The primary objective was to evaluate clinical, psychological, and sexual health parameters in cisgender women with endometriosis.

### 2.1. Participants

Eligible participants were cisgender women aged ≥ 18 years with a clinical diagnosis of endometriosis, recruited from the outpatient clinic and surgical unit. Exclusion criteria were pregnancy, menopause, and significant physical or psychiatric conditions that could independently impact quality of life, such as active malignancy, severe autoimmune disease, major depressive disorder, psychotic disorders, or neurological conditions affecting pain or sexual function.

To ensure clarity regarding disease timeline, the time since diagnosis was recorded for each participant. Most participants had received their diagnosis more than four weeks prior to the study, ensuring that reported sexual function reflected their lived experience with the disease rather than an acute diagnostic phase.

All participants provided written informed consent before enrollment. A total of 300 patients were screened, of whom 102 completed both a physical examination and an online questionnaire and were included in the final analysis. Seventy-seven had histologically confirmed endometriosis following surgery; 25 had no surgical intervention. Hormonal therapy was ongoing in 35 participants. Surgical versus non-surgical management was determined based on clinical evaluation, imaging findings, symptom severity, and shared decision-making with their physician. The sample size was determined based on the population of our specialized endometriosis consultation. Data collection was conducted over one year to ensure an adequate representation of patients attending the consultation within this period. Initially, the study population consisted of 300 patients; however, only 102 participants fully completed the questionnaires, which represents a limitation of this study. This reduced sample size may impact the statistical power and the generalizability of the findings. Despite this limitation, the study provides valuable insights into the relationship between deep infiltrating endometriosis (DIE) and sexual function.

### 2.2. Procedures

All participants underwent standard gynecological examination. Operative reports were reviewed for those who underwent surgery (n = 77). Disease was classified using both the revised American Society for Reproductive Medicine (rASRM) and Enzian classification systems. Ethical approval was granted by the Faculty of Psychology and Educational Science and the ethics committee of Centro Hospitalar Universitário de Santo António (Ref. 188/19). Written informed consent was obtained from all participants prior to inclusion.

### 2.3. Instruments

#### 2.3.1. Sociodemographic and Clinical Data

A structured questionnaire collected data on age, education, occupation, relationship status, cohabitation, and parity.

#### 2.3.2. Endometriosis Classification

The rASRM system stages endometriosis from Stage I (minimal) to Stage IV (severe) based on lesion size, adhesions, and tubal involvement [11]. The Enzian classification provides a detailed anatomical mapping of deep infiltrating endometriosis (DIE), covering compartments A (vagina/rectovaginal space), B (uterosacral ligaments, pelvic wall), and C (rectum), as well as optional sites: F (bladder/ureters), O (ovaries), P (peritoneum), FI (intestinal), and T (tubal adhesions) [14].

#### 2.3.3. Sexual Function

The Female Sexual Function Index (FSFI) is a 19-item validated tool evaluating desire, arousal, lubrication, orgasm, satisfaction, and pain during the previous four weeks. Scores ≤ 26.55 indicate sexual dysfunction [17,32,33,34]. The FSFI has shown excellent internal consistency (Cronbach’s α = 0.82–0.97 across domains) [17,33,34]. An example item is “Over the past 4 weeks, how often did you feel sexual desire or interest?” (rated on a 5-point scale from “almost never or never” to “almost always or always”).

#### 2.3.4. Anxiety and Depression

The Hospital Anxiety and Depression Scale (HADS) consists of 14 items (7 each for anxiety and depression). Scores ≥ 11 suggest probable clinical disorder. Somatic symptoms are excluded to reduce confounding [35,36,37,38,39,40]. The HADS demonstrates good internal consistency (Cronbach’s α typically between 0.80 and 0.93 for anxiety and depression subscales) [39,40]. An example item is “I feel tense or ‘wound up’” (rated from 0 = “not at all” to 3 = “most of the time”).

#### 2.3.5. Perceived Stress

The Portuguese version of the Perceived Stress Scale (PSS-13) includes 13 items scored from 0 (“never”) to 4 (“very often”), with higher scores indicating more stress [41,42]. The PSS has demonstrated good reliability (Cronbach’s α = 0.83) in previous studies [41]. An example item is “In the last month, how often have you felt that you were unable to control the important things in your life?”

#### 2.3.6. Relationship Satisfaction

The Relationship Assessment Scale (RAS) consists of 7 items rated 1–5, with total scores from 7 to 35. Higher scores indicate greater relationship satisfaction [31]. The RAS typically demonstrates Cronbach’s α between 0.86 and 0.90 [31]. An example item is “How well does your partner meet your needs?” (rated from 1 = “poorly” to 5 = “extremely well”).

#### 2.3.7. Quality of Life

The Endometriosis Health Profile-30 (EHP-30) assesses five domains: pain, control/powerlessness, emotional well-being, social support, and self-image. Scores range from 0 (best) to 100 (worst) [43,44]. The EHP-30 has shown excellent internal consistency across domains (Cronbach’s α = 0.83–0.96) [43,44]. An example item is “During the last 4 weeks, how often did pain interfere with your daily activities?”

#### 2.3.8. Pain Intensity

Pain was measured using a 10-cm Visual Analog Scale (VAS), ranging from “no pain” to “worst imaginable pain.”

### 2.4. Statistical Analysis

Data were analyzed using IBM SPSS Statistics for Windows, Version 20.0 (IBM Corp., Armonk, NY, USA). Descriptive statistics were reported as means, standard deviations, or medians where appropriate. Group comparisons were performed using Fisher’s exact test, Mann–Whitney U test, or Student’s *t*-test. Multivariate analysis was conducted using backward logistic regression. Statistical significance was set at *p* < 0.05.

### 2.5. Data Availability

Data supporting the findings of this study are available from the corresponding author upon reasonable request. Restrictions apply to the availability of certain clinical data due to ethical and privacy considerations.

## 3. Results

### 3.1. Descriptive Characteristics

Table 1 summarizes the sociodemographic and clinical characteristics of the 102 participants. No sociodemographic or clinical variables were significantly associated with sexual dysfunction (FSFI ≤ 26.55).

The mean age was 37 years (SD ± 7; range: 23–56). Most participants had completed secondary education (59%), and 41% held higher education degrees. Regarding living arrangements, 43.2% cohabited with a partner, 33.3% lived with a partner and children, 15.7% with parents, and 7.8% with others. Overall, 82.4% were in a committed relationship; while 17.6% were single, divorced, or widowed. All participants reported engaging in sexual activity at least once in the previous four weeks. 

With respect to parity, 54.9% had one child, 42.2% had two or more, and 2.9% were nulliparous. The most common reason for consultation was pelvic pain (61.8%), followed by pain with abnormal bleeding (8.8%), ultrasound findings (8.8%), suspected bowel endometriosis (5.9%), infertility (3.9%), pain with infertility (2.9%), bleeding alone (4.9%), and umbilical endometriosis (3%).

The mean age at menarche was 12 years (SD ± 2), and 80.4% reported menstrual cycles lasting ≤7 days. Hormonal contraception was reported by 34.3%.

Sexual dysfunction was identified in 5.9% of participants (n = 6), the remaining 94.1% scored above the dysfunction threshold.

### 3.2. Disease Localization and Sexual Dysfunction

Among the 77 participants who underwent surgery, disease classification was performed using both the Enzian and rASRM systems (Table 1).

ENZIAN Classification: Compartment A (vagina/rectovaginal space): All patients with lesions (n = 4) reported sexual dysfunction; none without lesions did; compartment B (uterosacral ligaments/pelvic sidewall): All affected patients (n = 4) reported dysfunction; 68.5% without B involvement had no dysfunction; compartment C (rectum): No significant association.

rASRM Staging: No significant association was observed between rASRM stage and sexual dysfunction (*p* = 0.572; U = 122.5; r = 0.06).

Key Finding: Lesions in Enzian compartment B were associated with sexual dysfunction (Fisher’s exact test, *p* = 0.013 **; φ = 0.29), indicating a specific anatomical influence on sexual health.

### 3.3. Psychosocial Factors and Sexual Function

Table 2 explores the relationships between sexual function (as measured by FSFI), psychosocial variables, and disease classification (classified by ENZIAN and rASRM classifications).

Anxiety and Depression: No statistically significant associations were found between HADS scores and sexual dysfunction. Surprisingly, higher anxiety levels were observed among patients without sexual dysfunction, though this difference was not statistically significant.

Perceived Stress (PSS): Stress levels were not significantly associated with sexual dysfunction, surgical history, hormonal therapy, or disease classification.

Relationship Satisfaction: No significant associations were observed between RAS scores and sexual function.

Quality of Life (EHP-30): Sexual dysfunction was significantly associated with poorer quality of life, as assessed by the EHP-30. Notable differences were observed in the main questionnaire (*p* = 0.018 *; t(100) = −2.4; d = −1.01) and emotional well-being domain (*p* = 0.013 *; t(100) = −2.5; d = −1.06).

Additionally, patients who had undergone surgery reported significantly lower self-image scores (*p* = 0.012 *; t(100) = 2.56; d = 0.59). Pain scores were significantly lower in patients with Enzian A localization (*p* = 0.047 **; t(73.8) = 2.016; d = 0.44), though no significant differences were observed across Enzian B/C or rASRM classifications.

### 3.4. Symptoms and Sexual Function

Table 3 details associations between endometriosis-related symptoms (fatigue, chronic pelvic pain, dysmenorrhea, dyspareunia, dyschezia, and dysuria) and sexual dysfunction (assessed using the FSFI), and the anatomical distribution and extent of disease, as classified by the ENZIAN and rASRM systems, respectively.

Pain, measured via VAS, was significantly lower in patients with sexual dysfunction (mean = 4 ± 3) compared to those without dysfunction (mean = 7 ± 3; *p* = 0.024 *; t(99) = −2.3; d = −1.05). No significant associations were found between pain intensity and disease localization. Notably, a statistically significant inverse correlation was found between VAS pain and anxiety (*p* = 0.025 *; r = −0.22) (Table 4), suggesting that higher pain levels were paradoxically associated with lower anxiety. Depression scores did not correlate with pain (*p* = 0.788; r = −0.027).

No statistically significant associations were observed between symptom severity and either ENZIAN or rASRM classifications. However, participants with severe dysmenorrhea and chronic pelvic pain reported higher global pain scores.

### 3.5. Predictive Model

A backward logistic regression model explained 28.1% of the variance in sexual dysfunction (R ≤ 0.281). However, no individual predictors remained statistically significant in the final model (Table 5).

### 3.6. Summary of Key Findings

Enzian B localization was associated with sexual dysfunction (*p* = 0.013 *). Participants with sexual dysfunction reported lower overall pain scores (*p* = 0.024 *). Quality of life, particularly emotional well-being (EHP-30), was significantly worse among those with sexual dysfunction (*p* = 0.013 *). No other psychosocial or anatomical variables showed significant associations.

## 4. Discussion

This study explored the multifactorial impact of endometriosis on sexual function integrating anatomical, psychosocial, and quality of life factors. The findings underscore the complex interplay between lesion localization, pain perception, and emotional well-being in shaping sexual health outcomes.

Sexual dysfunction was identified in 5.9% of participants, a prevalence that is substantially lower than the 50–75% reported in previous studies [45]. This discrepancy may reflect the characteristics of a tertiary care setting, where patients typically receive earlier diagnosis, individualized treatment, and multidisciplinary support—factors that may contribute to better sexual function outcomes.

The low prevalence observed in this cohort may also result from the inclusion criterion of recent sexual activity, which may have excluded individuals experiencing severe dyspareunia or psychological distress, who are less likely to engage in intercourse. This could have underrepresented the most affected individuals, leading to an underestimation of sexual dysfunction prevalence. Additionally, the tertiary care setting may introduce selection bias, favoring patients with better-managed disease, earlier-stage diagnoses, or greater access to comprehensive care. Moreover, sexual dysfunction remains a sensitive and potentially stigmatized topic. The reliance on self-reported measures may result in underreporting due to social desirability bias or discomfort disclosing sexual symptoms.

Surgical intervention, particularly excision of DIE, has been shown to significantly reduce dyspareunia and improve sexual function. Similarly, medical therapies such as progestins, GnRH analogs, and combined oral contraceptives are effective in reducing pain symptoms, which may mitigate the negative impact of endometriosis on sexual health.

Additionally, patients who seek care at specialized centers may differ from those in community-based settings. They are often more proactive in symptom management, have greater access to expert care, and may have been diagnosed earlier in the disease course, leading to better overall outcomes. In contrast, studies reporting higher rates of sexual dysfunction frequently include populations where endometriosis is undiagnosed or inadequately treated, and where chronic pain remains uncontrolled, exacerbating sexual health impairments.

The low incidence of sexual dysfunction observed in our study suggests that comprehensive treatment strategies implemented in a tertiary setting may significantly reduce the burden of sexual dysfunction in patients with endometriosis. However, further research is warranted to assess the long-term impact of different therapeutic approaches, particularly in non-specialized settings, to better understand the role of early intervention and continuous management in preserving sexual function.

Notably, while the multivariable logistic regression model explained 28.1% of the variance in sexual dysfunction, no individual variables remained statistically significant. This likely reflects the small number of participants meeting criteria for dysfunction (n = 6), which limits the model’s statistical power. With so few outcome events, the regression estimates become unstable, and even true associations may not reach significance. Additionally, the inclusion criterion requiring recent sexual activity may have excluded those with more severe dysfunction or pain, skewing the sample toward less impaired individuals and reducing the variability necessary to detect associations. The tertiary care setting may also contribute to a more homogeneous cohort with better symptom control, limiting the contrast between groups. Together, these factors likely contributed to the lack of significant predictors in the final model, despite its moderate explanatory power.

### 4.1. Disease Localization and Sexual Dysfunction

Lesions in Enzian B compartments (sacrouterine ligaments, cardinal ligaments, pelvic sidewall) were associated with sexual dysfunction, whereas rASRM stage was not. This reinforces critiques of the rASRM system’s limitations in capturing DIE-associated pain [12]. The Enzian system allowed for more precise anatomical mapping and better correlation with clinical outcomes, supporting its routine use in evaluating endometriosis-related sexual dysfunction.

The concurrent use of the rASRM and Enzian classification systems may represent an important strength in both clinical and research settings. The rASRM system provides a standardized and widely recognized framework for staging endometriosis but has limited capacity to capture the anatomical complexity of deep infiltrating endometriosis (DIE). In contrast, the Enzian system offers detailed compartmental mapping of lesions involving structures such as the uterosacral ligaments, vagina, bladder, and bowel, allowing for better anatomical–clinical correlation. By combining both systems, clinicians and researchers can achieve a more comprehensive characterization of disease burden, improve patient stratification, and better predict pain and sexual function outcomes. However, both systems have limitations: neither fully accounts for extra-pelvic disease, central sensitization, or psychosocial contributors, which can strongly influence patient experience. Future classification frameworks could benefit from integrating symptom severity and psychosocial domains to provide a more holistic representation of disease impact.

This study highlights the novel application of the Enzian classification in the context of sexual function, offering a more anatomically sensitive framework for understanding symptom localization and its impact on patient-reported outcomes.

### 4.2. Pain and Sexual Dysfunction

Paradoxically, participants with sexual dysfunction reported lower VAS pain scores. This may reflect the study’s inclusion criteria, which required recent sexual activity and could have excluded individuals with more severe pain or central sensitization. As such, the sample may be skewed toward individuals with sufficient physical capacity to remain sexually active despite distress.

Pain perception is shaped by both biological and psychosocial factors, including central sensitization and pelvic floor dysfunction [10]. These mechanisms may not directly reflect lesion burden but still contribute to sexual impairment. No association between lesion localization and pain intensity was found, highlighting the role of central pain modulation and behavioral adaptation.

This paradoxical relationship may also reflect the multifaceted nature of sexual dysfunction, in which low FSFI scores can occur in the absence of high pain levels. Psychological and relational dimensions—such as reduced libido, emotional disconnection, or partner-related stress—may negatively influence sexual function independently of nociceptive input. In some cases, women with less severe pain may still experience sexual distress due to unresolved emotional factors, communication difficulties, or performance anxiety. These findings underscore the importance of interpreting FSFI outcomes within a broader biopsychosocial framework, recognizing that sexual dysfunction is not solely a function of physical pain but is shaped by complex interpersonal and psychological dynamics.

### 4.3. Psychological Factors and Sexual Function

Contrary to prior findings [19,20,21,22,23], no significant associations were found between sexual dysfunction and anxiety, depression, or stress. Interestingly, higher anxiety scores were observed in participants without sexual dysfunction, possibly reflecting adaptive coping mechanisms or psychological resilience. The inverse correlation between pain intensity and anxiety (*p* = 0.025) raises questions about pain processing and its dissociation from sexual function. Future research should explore protective traits such as resilience, emotional regulation, and the use of tools like the Female Sexual Distress Scale (FSDS) to better assess the emotional burden of dysfunction [46].

### 4.4. Relationship Satisfaction and Sexual Health

Despite known relational impacts of endometriosis [24,25,26,27,28,29], relationship satisfaction was not significantly associated with sexual function. This aligns with studies showing that sexual satisfaction can persist despite dysfunction [47], suggesting that emotional intimacy and communication may buffer against physical limitations. Our cohort may include resilient couples who have adapted well, especially within a supportive care context.

### 4.5. Quality of Life and Sexual Dysfunction

Sexual dysfunction was significantly associated with reduced quality of life, particularly emotional well-being (*p* = 0.045 *). Lower self-image scores in surgical patients (*p* = 0.012 *) may reflect the psychological toll of repeated interventions. These findings reinforce the need for integrated approaches addressing both physical and emotional aspects of care.

### 4.6. Strengths and Limitations

Strengths include the use of the Enzian classification and validated instruments assessing sexual, psychological, and quality of life domains. The tertiary care setting enabled insight into actively managed patients. The combined use of Enzian and rASRM represents an innovative aspect of this study, allowing a more nuanced anatomical understanding of sexual dysfunction in endometriosis. Notably, the application of the Enzian classification to explore associations with sexual function represents a novel and promising approach to understanding the anatomical correlates of symptom severity in endometriosis.

Limitations include potential selection bias, a low number of participants meeting the dysfunction threshold (n = 6), and a cross-sectional design that limits causal inference. The inclusion criterion requiring recent sexual activity may have unintentionally excluded women with more severe pain, who are often less likely to engage in sexual activity due to discomfort or avoidance. This exclusion may have led to an underrepresentation of those with the most significant impairments in sexual function, potentially biasing the results toward less severe cases. Furthermore, the small number of participants meeting criteria for sexual dysfunction (n = 6) substantially limits the statistical power to detect meaningful associations or differences. This low case number reduces the robustness and generalizability of conclusions drawn from subgroup analyses. To enhance interpretability despite these constraints, we reported effect size measures (Cohen’s d, r, and φ) for all significant and non-significant results, allowing evaluation of the magnitude and practical relevance of observed effects independently of sample size. Nevertheless, the limited sample size may have reduced sensitivity to detect small effects, and findings should therefore be considered preliminary. Additionally, the use of self-reported measures—particularly for sensitive topics such as sexual health, psychological distress, and relationship satisfaction—may introduce reporting bias due to factors such as social desirability or recall inaccuracies.

### 4.7. Future Directions

Overall, this study underscores the importance of integrating anatomical classification systems with psychosocial assessment to better understand the multifactorial nature of sexual dysfunction in endometriosis. Future research should focus on refining classification tools to include not only lesion localization and extent but also symptom severity, pain mechanisms, and psychological well-being, ultimately allowing for more tailored and effective patient care.

## 5. Conclusions

This study underscores the multifactorial nature of sexual dysfunction in women with endometriosis and highlights lesion localization, particularly in the parametrial region (Enzian B), as a significant anatomical contributor. These findings reaffirm the limitations of rASRM staging and support the clinical relevance of the Enzian classification.

Although pain emerged as a key factor, lower scores among those with dysfunction suggest that sexual outcomes are shaped by more than symptom severity. While psychological and relational factors were not statistically significant in this sample, they remain important domains warranting further exploration. These findings advocate for a multidisciplinary, patient-centered approach, integrating anatomical, psychological, and relational care to preserve sexual health and improve quality of life in women with endometriosis. Future studies should adopt longitudinal designs, explore central sensitization mechanisms, and investigate targeted interventions to improve sexual health and quality of life.

## Figures and Tables

**Table 1:** Sociodemographic and biomedical characteristics of the participants and sexual dysfunction (as measured by FSFI), disease localization (as classified by ENZIAN) and disease extent (as classified by rASRM).

Sociodemographic and Biomedical Characteristics of the Participants, Sexual Dysfunction, Disease Localization and Disease Extent					
Characteristics	Total	Sexual Dysfunction		*p*-Value	Effect Size Measures
		Yes	No		
Age (years)	37 ± 7 [23; 56]	41 ± 11 [27; 56]	36 ± 6 [23; 53]	0.141	t(74) = 1.49; d = 0.68
Educational qualifications					φ = 0.046
Secondary education					
Higher education	60 (59%)	3 (50%)	57(59.4%)	0.687	
	42 (41%)	3 (50%)	39(40.6%)		
Lives with					
Spouse or partner	44 (43.2%)	0 (0%)	44 (45.8%)	NA (1)	
Spouse or partner and children or stepchildren	34 (33.3)%	2 (33.3%)	32 (33.3%)		
Parents	16 (15.7%)	2 (33.3%)	14 (14.6%)		
Other	8 (7.8%)	2 (33.3%)	6 (6.3%)		
Relational Status					φ = 0.212
Married or registered partnership + Committed relationship	84 (82.4%)	3 (50%)	81 (84.4%)	0.066	
Single, divorced, widow	18 (17.6%)	3 (50%)	15 (15.6%)		
					t(100) = 0.716; d = 0.30
	3 (2.9%)	3 (50%)	53(55.2%)	0.475	
	56 (54.9%)	1(16.7%)	28(29.2%)		
	43 (42.2%)	2(33.3%)	15(15.6%)		
Reason for Consultation					
Pain	63 (61.8%)	3 (100%)	60 (62.5%)	NA (1)	
Pain + Infertility	3 (2.9%)	0 (0%)	3 (3.1%)		
Pain + Abnormal uterine bleeding	9 (8.8%)	0 (0%)	9 (9.4%)		
Infertility	4 (3.9%)	0 (0%)	4 (4.2%)		
Abnormal uterine bleeding	5 (4.9)%	0 (0%)	2 (2.1%)		
Endometriosis lesions on ultrasound	9 (8.8)%	0 (0%)	9 (9.4%)		
Sent by general surgery or gastroenterology (endometriosis identified on the bowel)	6 (5.9%)	0 (0%)	6 (6.3%)		
Endometriosis nodule on the umbilicus	3 (3%)	0 (0%)	3 (3.1%)		
Age at Menarche (year)	12 ± 2 [8; 16]	14 ± 1 [13; 14]	12 ± 2 [8; 16]	0.057	t(57) = 1.94; d = 1.01
Duration of menses					φ = 0.12
≤7 days	82 (80.4%)	3(100%)	29(85.3%)	1.000	
≥8 days	20 (19.6%)	0 (0%)	5 (14.7%)		
Contraception used					φ = 0.083
Yes	35 (34.3%)	3 (50%)	32(33.3%)	0.336	
No	67 (65.7%)	3 (50%)	64(66.7%)		
Enzian A					φ = 0.192
No	31 (42.5)	0 (0)	31(42.5)	0.144	
Yes	44 (57.5)	4 (100)	42 (57.5)		
Enzian B					φ = 0.29
No	50 (64.9)	0 (0)	50 (68.5)	0.013 *	
Yes	27 (35.1)	4 (100)	23 (31.5)		
Enzian C					φ = 0.005
No	57 (74)	3 (75)	54 (74)	1	
Yes	20 (26)	1 (25)	19 (26)		
rASRM Score	3 [2; 4]	2 [1; 4]	3 [3; 4]	0.572	U = 122.5; r = 0.06
rASRM					φ = 0.12
I, II	39 (50.6)	1 (25)	38 (52.1)	0.358	
III, IV	38 (49.4)	3 (75)	35 (47.9)		

Data are presented as mean ± standard deviation, median [IQR], or n (1). A *p*-value could not be calculated because the assumptions of the Chi-square test were not met (i.e., more than 20% of the cells had expected counts less than 5). * Statistically significant (*p*-value < 0.05).

**Table 2:** Relationship between psychosocial factors, sexual dysfunction, (as measured by FSFI), disease localization (as classified by ENZIAN) and disease extent (as classified by rASRM).

Relationship Between Psychosocial Factors, Sexual Dysfunction, Disease Localization and Disease Extent				
Anxiety and Depression and Sexual Dysfunction				
	Sexual Dysfunction		*p*-Value	Effect Size Measures
	No	Yes		
Score Anxiety	17.56 ± 2.61	19.33 ± 2.58	0.109	t(100) = 1.615; d = 0.68
Score Depression	16.09 ± 1.82	15.67 ± 1.03	0.571	t(100) = −0.568; d = −0.23
**Anxiety and Depression and Disease Localization**				
	**Enzian A**		***p*** **-Value**	
	**No**	**Yes**		
Score Anxiety	17.68 ± 2.89	17.63 ± 2.40	0.938	t(75) = 0.078; d = 0.02
Score Depression	15.68 ± 1.60	16.48 ± 1.96	0.063	t(75) = −1.887; d = −0.44
	**Enzian B**		***p*** **-Value**	
	**No**	**Yes**		
Score Anxiety	17.58 ± 2.47	17.78 ± 2.85	0.751	t(75) = −0.318; d = −0.076
Score Depression	15.96 ± 1.37	16.52 ± 2.52	0.292	t(75) = −1.264; d = −0.30
	**Enzian C**		***p*** **-Value**	
	**No**	**Yes**		
Score Anxiety	17.58 ± 2.58	17.85 ± 2.68	0.690	t(75) = −0.401; d = −0.10
Score Depression	15.96 ± 1.88	16.70 ± 1.72	0.129	t(75) = −1.537; d = −0.40
**Anxiety and Depression and Disease Extent**				
	**rASRM**		***p*** **-Value**	
	**I, II**	**III, IV**		
Score Anxiety	17.46 ± 2.5	17.84 ± 2.70	0.523	t(75) = −0.642; d = −0.15
Score Depression	16.31 ± 1.96	16 ± 1.76	0.471	t(75) = 0.725; d = 0.17
**Perceived Stress and Sexual Dysfunction**				
	**Sexual Dysfunction**		***p*** **-Value**	
	**No**	**Yes**		
Perceived stress	41.49 ± 4.55	40.17 ± 4.67	0.492	t(98) = −0.690; d = −0.29
**Perceived Stress and Localization of the Disease**				
Perceived stress	**Enzian A**		***p*** **-Value**	
	**No**	**Yes**		
	42.17 ± 4.62	40.85 ± 4.79	0.238	t(74) = 1.189; d = −0.28
	**Enzian B**		***p*** **-Value**	
	**No**	**Yes**		
	41.70 ± 4.53	40.73 ± 5.14	0.401	t(74) = 0.844; d = −0.20
	**Enzian C**		***p*** **-Value**	
	**No**	**Yes**		
	41.52 ± 4.85	40.95 ± 4.51	0.649	t(74) = 0.458; d = −0.12
**Perceived Stress and Disease Extent**				
	**rASRM**		***p*** **-Value**	
	**I, II**	**III, IV**		
Perceived stress	40.76 ± 4.08	41.97 ± 5.30	0.268	t(74) = −1.115; d = −0.26
**Relationship Satisfaction and Sexual Dysfunction**				
	**Sexual Dysfunction**		***p*** **-Value**	
	**No**	**Yes**		
Relationship satisfaction	24.24 ± 4.40	22.67 ± 4.46	0.398	t(95) = −0.850; d = −0.36
**Relationship Satisfaction and Localization of the Disease**				
Relationship satisfaction	**Enzian A**		***p*** **-Value**	
	**No**	**Yes**		
	23.93 ± 3.87	23.50 ± 4.94	0.294	t(71) = −0.396; d = −0.10
	**Enzian B**		***p*** **-Value**	
	**No**	**Yes**		
	23.45 ± 4.63	24.08 ± 4.38	0.995	t(71) = −0.572; d = −0.14
	**Enzian C**		***p*** **-Value**	
	**No**	**Yes**		
	23.39 ± 4.60	24.27 ± 4.31	0.372	t(71) = −0.898; d = −0.24
**Quality of Life and Sexual Dysfunction**				
	**Sexual Dysfunction**		***p*** **-Value**	
	**No**	**Yes**		
Core questionnaire	54.5 ± 18.8	35.3 ± 21.4	0.018 *	t(100) = −2.4; d = −1.01
Additional modules				
Work	42.1 ± 23	33.6 ± 26.2	0.429	t(92) = −0.795; d = −0.017
Relationship with child/children	45.3 ± 22.8	50 ± 14.1	0.778	t(43) = 0.284; d = 0.009
Sexual relationship	63.7 ± 24.4	60 ± 56.6	0.942	t(86) = −0.205; d = −0.04
Feelings about medical profession	36.8 ± 22.1	31.7 ± 20.4	0.580	t(97) = −0.556; d = −0.11
Feelings about treatment	46 ± 20	34.8 ± 19.8	0.226	t(80) = −1.219; d = −0.27
Feelings about infertility	58.1 ± 28.5	30 ± 17.3	0.095	t(77) = −1.691; d = −0.36
Pain	51.3 ± 22.4	30.3 ± 22.1	0.028 *	t(100) = −2.227; d = −0.45
Control and powerlessness	55 ± 23.8	36.5 ± 28.3	0.071	t(100) = −1.825; d = −0.37
Emotional well-being	56.6 ± 19.5	35.7 ± 22.8	0.013 *	t(100) = −2.5; d = −1.06
Social support	56.5 ± 21.9	46.7 ± 26	0.293	t(100) = −1.058; d = −0.21
Self-image	57.3 ± 24.1	35.5 ± 14.2	0.031 *	t(100) = −2.188; d = −0.44
**Quality of Life and Disease Localization**				
	**Enzian A**		***p*** **-Value**	
	**No**	**Yes**		
Core questionnaire	56.9 ± 17.7	51.9 ± 19.8	0.267	t(75) = 1.119; d = −0.26
Additional modules				
Work	41.7 ± 23.4	41.9 ± 24.5	0.967	t(67) = −0.042; d = −0.01
Relationship with child/children	47.2 ± 20.5	43.8 ± 27	0.674	t(32) = 424; d = 0.15
Sexual relationship	65.9 ± 21.6	65.7 ± 26	0.975	t(66) = 0.032; d = 0.01
Feelings about medical profession	35.3 ± 21.1	34.5 ± 22	0.879	t(73) = 0.153; d = −0.04
Feelings about treatment	48 ± 20.8	45.4 ± 20.2	0.612	t(63) = 0.509; d = 0.13
Feelings about infertility	56.6 ± 31.1	57.5 ± 26.7	0.909	t(75) = −0.114; d = 0.03
Pain	55.9 ± 17.7	46.5 ± 23.3	0.047 *	t(73.8) = 2.02; d = 0.44
Control and powerlessness	56.1 ± 23.1	51.3 ± 24.4	0.386	t(75) = 0.873; d = 0.20
Emotional well-being	56.2 ± 20	56.3 ± 21.6	0.987	t(75) = −0.016; d = 0.00
Social support	56.1 ± 20.5	57.8 ± 21.4	0.730	t(75) = −0.347; d = 0.08
Self-image	63.8 ± 22.1	56.5 ± 24.5	0.182	t(75) = 1.347; d = 0.31
	**Enzian B**		***p*** **-Value**	
	**No**	**Yes**		
Core questionnaire	55.5 ± 18.7	51.1 ± 19.6	0.337	
Additional modules				t(75) = 0.965; d = 0.22
Work	41.6 ± 23.1	42.2 ± 25.7	0.926	t(67) = −0.09; d = −0.02
Relationship with child/children	45.4 ± 24.9	46.3 ± 20	0.929	t(32) = −0.099; d = 0.03
Sexual relationship	63.8 ± 22.3	69.5 ± 27.1	0.356	t(66) = −0.392; d = 0.10
Feelings about medical profession	36 ± 22.4	32.8 ± 20.1	0.531	t(73) = 0.629; d = 0.15
Feelings about treatment	45.9 ± 21.3	47.3 ± 18.8	0.797	t(63) = −0.259; d = −0.06
Feelings about infertility	53.9 ± 29.3	63.6 ± 26.6	0.215	t(59) = −1.254; d = −0.33
Pain	51.1 ± 21.8	48.8 ± 21.5	0.653	t(75) = 0.452; d = 0.10
Control and powerlessness	54 ± 23.5	51.8 ± 24.9	0.704	t(75) = 0.381; d = 0.09
Emotional well-being	59.2 ± 20.1	50.9 ± 21.5	0.098	t(75) = 1.677; d = 0.39
Social support	59.3 ± 20	53.1 ± 22.3	0.221	t(75) = 1.235; d = 0.28
Self-image	61.6 ± 24.7	55.3 ± 21.6	0.268	t(75) = 1.115; d = 0.26
	**Enzian C**		***p*** **-Value**	
	**No**	**Yes**		
Core questionnaire	55 ± 19.7	51 ± 17.1	0.420	t(75) = 0.811; d = 0.19
Additional modules				
Work	42.8 ± 23.9	38.8 ± 24	0.557	t(67) = 0.590; d = 0.14
Relationship with child/children	47.2 ± 23.2	41.1 ± 25.2	0.514	t(32) = 0.661; d = 0.23
Sexual relationship	66.4 ± 23.7	64.4 ± 25.5	0.759	t(66) = 0.309; d = 0.07
Feelings about medical profession	36.5 ± 23.1	30.5 ± 16.1	0.292	t(48.609) = 1.254; d = 0.36
Feelings about treatment	48.2 ± 20.6	41.2 ± 19.2	0.230	t(63) = 1.213; d = 0.30
Feelings about infertility	58.4 ± 29.2	53 ± 27.2	0.530	t(59) = 0.655; d = 0.17
Pain	51.2 ± 22.4	47.7 ± 19.5	0.527	t(75) = 0.635; d = 0.15
Control and powerlessness	54.7 ± 24.8	49 ± 21.1	0.354	t(75) = 0.932; d = 0.21
Emotional well-being	56.1 ± 21.5	56.9 ± 19.2	0.877	t(75) = −0.156; d = 0.04
Social support	58.9 ± 20.6	52.3 ± 21.7	0.227	t(75) = 1.219; d = 0.28
Self-image	61.4 ± 23.7	53.8 ± 23.6	0.215	t(75) = 1.245; d = 0.29
**Quality of Life and Disease Extent**				
	**rASRM**		***p*** **-Value**	
	**I. II**	**III. IV**		
Core questionnaire	56.4 ± 19.9	51.4 ± 18	0.257	t(75) = 1.142; d = 0.26
Additional modules				
Work	44.9 ± 23.4	38.4 ± 24.2	0.263	t(67) = 1.128; d = 0.28
Relationship with child/children	46.4 ± 21.5	44.2 ± 27.8	0.799	t(32) = 0.257; d = 0.06
Sexual relationship	71.4 ± 23.3	60.5 ± 23.8	0.062	t(66) = 1.900; d = 0.46
Feelings about medical profession	39.1 ± 24	30.8 ± 18.1	0.098	t(66.830) = 1.680; d = 0.41
Feelings about treatment	48 ± 20.4	44.5 ± 20.4	0.862	t(63) = 0.693; d = 0.17
Feelings about infertility	57.2 ± 29.2	56.9 ± 28.3	0.966	t(59) = 0.043; d = 0.01
Pain	51.1 ± 23.2	49.4 ± 20.1	0.735	t(75) = 0.339; d = 0.08
Control and powerlessness	56.1 ± 25.1	50.3 ± 22.4	0.297	t(75) = 1.05; d = 0.24
Emotional well-being	60.5 ± 20.9	51.9 ± 20.1	0.071	t(75) = 1.828; d = 0.42
Social support	60.3 ± 21.7	53.9 ± 19.9	0.188	t(75) = 1.330; d = 0.31
Self-image	62.3 ± 25.4	56.5 ± 21.8	0.288	t(75) = 1.071; d = 0.25
**Quality of Life and Taking Hormonal Therapy**				
	**Hormonal Therapy**		***p*** **-Value**	
	**No**	**Yes**		
Core questionnaire	53.3 ± 19.1	53.4 ± 20.1	0.980	t(92) = 0.071; d = 0.01
Additional modules				
Work	41.5 ± 23.2	41.9 ± 23.4	0.943	t(92) = 0.071; d = 0.01
Relationship with child/children	47.4 ± 23.5	41.4 ± 19.9	0.413	t(43) = −0.827; d = −0.25
Sexual relationship	61.6 ± 24.1	67.7 ± 26.4	0.278	t(86) = 1.092; d = 0.23
Feelings about medical profession	37.3 ± 22.3	34.8 ± 21.4	0.604	t(97) = −0.521; d = −0.12
Feelings about treatment	44.5 ± 20.5	46.7 ± 19.5	0.644	t(64) = 0.464; d = 0.11
Feelings about infertility	57.8 ± 29.6	55.3 ± 26.5	0.726	t(77) = −0.352; d = 0.08
Pain	50.3 ± 22.2	49.6 ± 24.2	0.874	t(100) = −0.158; d = −0.03
Control and powerlessness	53.8 ± 24.6	54.2 ± 24.3	0.932	t(100) = 0.086; d = 0.02
Emotional well-being	54.8 ± 19.7	56.4 ± 21.5	0.700	t(100) = 0.387; d = 0.08
Social support	56.2 ± 22.6	55.4 ± 21.4	0.869	t(100) = −0.165; d = −0.03
Self-image	55.6 ± 24.5	56.8 ± 23.9	0.819	t(100) = 0.229; d = 0.05
**Quality of Life and Being Submitted to Surgery**				
	**Surgery**		***p*** **-Value**	
	**No**	**Yes**		
Core questionnaire	51.5 ± 20.8	53.9 ± 19	0.593	t(100) = 0.537; d = 0.12
Additional modules				
Work	41.1 ± 21.5	41.8 ± 23.8	0.901	t(92) = 0.125; d = 0.19
Relationship with child/children	45.5 ± 19.7	45.6 ± 23.5	0.987	t(43) = 0.017; d = 0.30
Sexual relationship	56 ± 26.9	65.8 ± 24.1	0.122	t(86) = 1.563; d = 0.32
Feelings about medical profession	41.7 ± 23.2	34.9 ± 21.5	0.188	t(97) = −1.325; d = −0.04
Feelings about treatment	41.5 ± 19	46.4 ± 20.3	0.380	t(80) = 0.883; d = 0.08
Feelings about infertility	56.9 ± 29.4	57.1 ± 28.6	0.988	t(77) = 0.016; d = 0.00
Pain	49.4 ± 26.7	50.3 ± 21.6	0.859	t(78) = 0.178; d = 0.19
Control and powerlessness	56 ± 26.3	53.2 ± 23.9	0.624	t(100) = −0.491; d = 0.12
Emotional well-being	52.4 ± 18.4	56.3 ± 20.8	0.408	t(100) = 0.831; d = 0.08
Social support	52.2 ± 25.6	57.1 ± 20.9	0.387	t(35.037) = 0.876; d = 0.12
Self-image	45.6 ± 22.9	59.4 ± 23.7	0.012 *	t(100) = 2.56; d = 0.59

Data are presented as mean ± standard deviation. * Statistically significant (*p*-value < 0.05).

**Table 3:** Relationship between disease symptoms, sexual dysfunction (as measured by FSFI), disease localization (as classified by ENZIAN) and disease extent (as classified by rASRM).

Relationship Between Disease Symptoms, Sexual Dysfunction and Disease Localization and Extent				
Pain Intensity and Sexual Dysfunction				
	Sexual Dysfunction		*p*-Value	Effect Size Measures
	No	Yes		
Pain intensity	7 ± 3	4 ± 3	0.024 *	t(99) = −2.3; d = −1.05
**Pain Intensity and Localization of the Disease**				
Pain intensity	**Enzian A**		***p*** **-Value**	
	**No**	**Yes**		
	7 ± 3	6 ± 3	0.321	t(74) = 1; d = 0.23
	**Enzian B**		***p*** **-Value**	
	**No**	**Yes**		
	6 ± 3	7 ± 3	0.455	t(74) = −0.751; d = −0.17
	**Enzian C**		***p*** **-Value**	
	**No**	**Yes**		
	7 ± 3	6 ± 3	0.890	t(43.44) = 0.139; d = 0.04
	**rASRM**		***p*** **-Value**	
	**No**	**Yes**		
	7 ± 3	6 ± 3	0.486	t(74) = 0.701; d = 0.16
**Tiredness or Lack of Energy, Localization of the Disease and Disease Extent**				
Tiredness/Lack of energy	**Enzian A**		***p*** **-Value**	
	**No**	**Yes**		
	8 (32%)	17 (68%)	0.305	φ = 0.12
	**Enzian B**		***p*** **-Value**	
	**No**	**Yes**		
	17 (68%)	8 (32%)	0.696	φ = 0.045
	**Enzian C**		***p*** **-Value**	
	**No**	**Yes**		
	18 (72%)	7 (28%)	0.779	φ = 0.032
	**rASRM**		***p*** **-Value**	
	**I, II**	**III, IV**		
	13 (52%)	12 (48%)	0.869	φ = 0.019
**Dysmenorrhea, Localization of the Disease and Disease Extent**				
Dysmenorrhea	**Enzian A**		***p*** **-Value**	
	**No**	**Yes**		
	7.1 ± 3.8	5.1 ± 4.2	0.132	t(39) = 1.537; d = 0.51
	**Enzian B**		***p*** **-Value**	
	**No**	**Yes**		
	5.6 ± 4.1	6.1 ± 4.3	0.677	t(39) = −0.420; d = 0.13
	**Enzian C**		***p*** **-Value**	
	**No**	**Yes**		
	5.1 ± 4	8 ± 3.9	0.051	t(39) = −2.015; d = 0.73
	**rASRM**		***p*** **-Value**	
	**I, II**	**III, IV**		
	6.3 ± 4.1	5.2 ± 4.2	0.421	t(39) = 0.813; d = 0.25
**Dyspareunia, Localization of the Disease and Disease Extent**				
**Dyspareunia**	**Enzian A**		***p*** **-Value**	
	No	Yes		
	3.6 ± 3.1	5.3 ± 3.7	0.105	t(47) = −1.653; d = 0.48
	**Enzian B**		***p*** **-Value**	
	**No**	**Yes**		
	4.1 ± 3.4	5.6 ± 3.8	0.165	t(47) = −1.412; d = 0.42
	**Enzian C**		***p*** **-Value**	
	**No**	**Yes**		
	4.2 ± 3.4	5.9 ± 3.8	0.132	t(47) = −1.534; d = 0.50
	**rASRM**		***p*** **-Value**	
	**I, II**	**III, IV**		
	5.2 ± 3.7	4 ± 3.4	0.241	t(47) = 1.187; d = 0.34
**Dyschesia, Localization of the Disease and Disease extent**				
Dyschesia	**Enzian A**		***p*** **-Value**	
	**No**	**Yes**		
	2.3 ± 3	2.6 ± 2.9	0.713	t(49) = −0.370; d = 0.11
	**Enzian B**		***p*** **-Value**	
	**No**	**Yes**		
	2.2 ± 2.7	3.1 ± 3.3	0.265	t(49) = −1.127; d = 0.33
	**Enzian C**		***p*** **-Value**	
	**No**	**Yes**		
	2.4 ± 3	2.7 ± 2.8	0.797	t(49) = −0.259; d = 0.08
	**rASRM**		***p*** **-Value**	
	**I, II**	**III, IV**		
	2.6 ± 3	2.4 ± 2.8	0.832	t(49) = 0.213; d = 0.06
**Dysuria, Localization of the Disease and Disease Extent**				
Dysuria	**Enzian A**		***p*** **-Value**	
	**No**	**Yes**		
	1.9 ± 2.9	1.5 ± 1.8	0.549	t(49) = 0.603; d = 0.18
	**Enzian B**		***p*** **-Value**	
	**No**	**Yes**		
	1.3 ± 1.9	2.1 ± 2.8	0.299	t(28.323) = −1.059; d = 0.34
	**Enzian C**		***p*** **-Value**	
	**No**	**Yes**		
	1.4 ± 1.7	2.5 ± 3.5	0.299	t(12.596) = −1.082; d = 0.51
	**rASRM**		***p*** **-Value**	
	**I, II**	**III, IV**		
	1.7 ± 2.7	1.5 ± 1.6	0.695	t(49) = 0.395; d = 0.11

Data are presented as mean ± standard deviation or n (%). * Statistically significant (*p*-value < 0.05).

**Table 4:** Relationship between anxiety and depression evaluated with HADS and pain intensity evaluated with VAS.

Relationship Between Anxiety and Depression and Pain Intensity		
Anxiety and Pain Intensity Evaluated with VAS		
	Anxiety	
	Rho	*p*-Value
Pain intensity	−0.222	0.025 *
**Depression and Pain Intensity**		
	**Depression**	
	**Rho**	***p*** **-Value**
Pain intensity	−0.027	0.788

* Statistically significant (*p*-value < 0.05).

**Table 5:** Hierarchical logistic regression, using sexual dysfunction (yes/no) as the dependent (outcome) variable.

Hierarchical Logistic Regression, Using Sexual Dysfunction as the Dependent Variable.				
	β	OR	CI 95%	*p*-Value
EHP Total	1.022	2.77	0.503–15.3	0.241
Enzian B (Yes)	25.940	1.84 × 10^11^	0–NA	0.994
Constant	−29.632	0	NA–NA	0.255

## Data Availability

The original contributions presented in the study are included in the article, further inquiries can be directed to the corresponding authors.
